# Connectome analysis of brain functional network alterations in breast cancer survivors with and without chemotherapy

**DOI:** 10.1371/journal.pone.0232548

**Published:** 2020-05-04

**Authors:** Vincent Chin-Hung Chen, Kai-Yi Lin, Yuan-Hsiung Tsai, Jun-Cheng Weng

**Affiliations:** 1 School of Medicine, Chang Gung University, Taoyuan, Taiwan; 2 Department of Psychiatry, Chang Gung Memorial Hospital, Chiayi, Taiwan; 3 Department of Medical Imaging and Radiological Sciences, Chang Gung University, Taoyuan, Taiwan; 4 Department of Diagnostic Radiology, Chang Gung Memorial Hospital, Chiayi, Taiwan; 5 Medical Imaging Research Center, Institute for Radiological Research, Chang Gung University and Chang Gung Memorial Hospital at Linkou, Taoyuan, Taiwan; University of North Carolina at Chapel Hill, UNITED STATES

## Abstract

**Purpose:**

Treatment modalities for breast cancer, the leading cause of cancer-related deaths in women worldwide, include surgery, radiotherapy, adjuvant chemotherapy, targeted therapy, and hormonal therapy. The advancement in medical technology has facilitated substantial reduction in breast cancer mortality. However, patients may experience cognitive impairment after chemotherapy. This phenomenon called chemotherapy-induced cognitive impairment (i.e., “chemobrain”) is common among breast cancer survivors. However, cognitive function deficits may exist before chemotherapy initiation. This study examined the functional network alterations in breast survivors by using resting-state functional magnetic resonance imaging (fMRI).

**Methods:**

We recruited 172 female participants and separated them into three groups: C+ (57 breast cancer survivors who had finished 3–12-month-long chemotherapy), C- (45 breast cancer survivors who had not undergone chemotherapy), and HC (70 participants with no breast cancer history). We analyzed mean fractional amplitudes of low-frequency fluctuation and graph theoretical topologies from resting-state fMRI and applied network-based analysis to portray functional changes among the three groups.

**Results:**

Among the three groups, the C- group demonstrated hyperactivity in the prefrontal cortex, bilateral middle temporal gyrus, right inferior temporal gyrus and right angular gyrus. Only the left caudate demonstrated significantly more hypoactivity in the C- group than in the C+ group. Graph theoretical analysis demonstrated that the brains of the C+ group became inclined toward regular networks and the brains of the C- group became inclined toward random networks.

**Conclusion:**

Subtle alterations were noted in the brain activity and networks of our cancer survivors. Moreover, functional network disruptions occurred regardless of chemotherapeutic agent administration.

## Introduction

Breast cancer is the most commonly diagnosed cancer and the leading cause of cancer-related deaths among women worldwide, accounting for approximately 25% of cancer cases and 15% of cancer-related deaths. In Taiwan, nearly 1000 per 100,000 female individuals were diagnosed with breast cancer [1, 2]. Treatments for breast cancer include surgery, radiotherapy, adjuvant chemotherapy, targeted therapy, and hormonal therapy. Of the many studies revealing that subtle brain activity changes occur in patients who have undergone chemotherapy, several have indicated that breast cancer survivors who have undergone chemotherapy may have cognitive impairment. Chemotherapy-induced cognitive impairment (also called “chemobrain”)—indicated by impaired cognitive functions such as memory, executive functions, processing speed, and reaction time abilities—is common among breast, lung, prostate, and ovarian cancer survivors who have received chemotherapeutic agents [3, 4].

Studies have analyzed the effects of chemotherapeutic agents on breast cancer survivors and noted that deficits in basic cognitive function frequently predate the beginning of chemotherapy [5, 6]. These studies have underlined the value of evaluating patients before chemotherapy. Without appropriate evaluation of pretreatment symptoms, these symptoms could erroneously be considered the side effects of chemotherapy.

Imaging methods are commonly applied in human brain studies. Blood oxygenation level-dependent functional magnetic resonance imaging (fMRI) has been widely used to explore cerebral function. fMRI, an efficient tool that can demonstrate functional changes in the human brain, can be separated into task-based fMRI and resting-state fMRI (rs-fMRI). Compared with task-based fMRI, rs-MRI simplifies the experimental design by instructing the patient to relax and clear the mind and does not require them to follow certain task instructions. Crucial physiological information extracted from rs-fMRI results includes the amplitude of low-frequency fluctuations (ALFF), which represents the intensity of spontaneous cerebral activity, which shares similarities with fluctuations in neurophysiological signal, and therefore can indicate the activation of certain cerebral areas. Functional imaging can aid in portraying the impacts of breast cancer and treatment.

Graph theoretical approaches are also ubiquitous in exploring network changes in the human brain and provide valuable perspectives on quantifying functional networks. Graph theoretical approaches consider cerebral subregions nodes and the connections between subregions edges. The brain is assessed as an efficient organ that communicates information with high efficiency and low cost [7]. The human brain network is considered to have small-world network properties, which indicate that the brain is organized into both local cliques and global integration. Changes in cognitive function could imply functional changes in the brain. Graph theoretical approaches have been applied in exploring Alzheimer disease, schizophrenia, and traumatic brain injury [8–10]. Alteration in topological parameters implies that illness may alter human brain connectivity and suggests that cognitive symptoms and functional deficits are the disturbance of the functional network [11].

Compared with task-based fMRI, relatively few studies of rs-fMRI have been reported in the field of chemobrain. The current study analyzed the functional brain changes in rs-fMRI images of breast cancer survivors and those without a breast cancer history. In a previous study, we focused on the differences between postchemotherapy patients and healthy individuals [12]. Here, we also included cancer survivors who did not undergo chemotherapy and hypothesized that functional connectome changes are larger in both the cancer survivor groups than in the control group.

## Patients and methods

### Participants

In our study, we hypothesize that the exposure to breast cancer has significant impact on cerebral functions. Given that no previous longitudinal studies addressed the brain changes in MRI scans of the breast cancer survivors within one year after diagnosis, the sample size will be calculated according to the previous cross-sectional study for breast cancer survivors. The effect sizes for reported differences in hippocampus volume between breast cancer survivors and normal controls were -0.75 for the left hippocampus, -0.58 for the right, and -0.81 for the left posterior lobe [13]. A smallest sample size of n = 25 is required for each group in this study to provide 80% power to detect difference in hippocampal volume between the breast cancer survivor group and control group based on a 2-sided test at the 5% significance level and effect size of -0.81. Previous studies on longitudinal study for female breast cancer patients show the dropout rates were 33% [14] and 21% [15]. The dropout rate in this study is estimated to be 25% so the 35 participants are planned to be recruited in each group.

In total, 172 female participants from Chiayi Chang Gung Memorial Hospital were recruited and divided into three groups: C+ (57 breast cancer survivors who had undergone chemotherapy), C- (45 breast cancer survivors who had not undergone chemotherapy), and HC (70 sex-matched individuals without a breast cancer history). The inclusion criteria of breast cancer patients included age 20 to 55 female with pathological proved primary breast cancer. The exclusion criteria of breast cancer patients included end-stage of the breast cancer, underwent treatment for other cancer, post radiation therapy before investigation, evidence of brain metastasis or other brain insults, previously diagnosed with neuropsychiatric disorder or substance used and unable to have a MRI scan. The same exclusion criteria were used for HC in addition to having no evidence of breast cancer. Each participant was assessed on neuropsychological scales, including the Patient Health Questionnaire-9 (PHQ-9) and Hospital Anxiety and Depression Scale (HADS). This study was approved by the Institutional Review Board of Chang Gung Memorial Hospital, Chiayi, Taiwan. (No. 104-5082B, 201700256B0, 201702027B0), and the written informed consent was obtained from all patients.

The Chinese version of the Patient Health Questionnaire (PHQ-9): The Patient Health Questionnaire-9 (PHQ-9) [16] was designed to screen depression symptoms over the preceding 2 weeks and used a 4-point scale ranging from 0 (not at all) to 3 (nearly every day). The range of the PHQ is from 0 to 27, and a higher score of the PHQ indicates more severe depression symptoms. The PHQ is reported to have high reliability. A reliable and valid for detecting MDD among Chinese primary care patients [17]. Hospital Anxiety and Depression Scale (HADS): The HADS was developed to examine anxiety symptoms and depression in people with physical health problems. The HADS contains 14 items, namely seven items for anxiety symptoms and seven items for depression and uses a 4-point scale. The HADS has been suggested to be a reliable tool with satisfactory psychometric reliability and validity [18]. The HADS has been widely used in studies for different patient population [19, 20].

### Functional MRI acquisition

Before the fMRI, all participants were requested to relax with their eyes closed and clear their minds while remaining alert during the scan. In all 172 participants, fMRI data were acquired using 3T MRI scanner (Verio, Siemens, Germany) at Chiayi Chang Gung Memorial Hospital. The rs-fMRI was performed in a gradient echo planner imaging sequence with the repetition time/echo time = 2000/30 ms, flip angle = 90°, number of excitations = 1, field of view = 220 × 220 mm^2^, matrix size = 64 × 64, and voxel size = 3.4 × 3.4 × 4 mm^3^, and number of axial images = 31 were acquired to cover the entire brain volume. Each rs-fMRI run contained 300 image volumes, and the total scan time was approximately 10 min.

### Functional image preprocessing

Functional MRI data preprocessing was performed using statistical parametric mapping 12 (SPM12; Wellcome Department of Cognitive Neurology, London, UK). Data were processed for slice-timing correction and then realigned to their first volume for motion correction. The results of six head motion parameters surpassed 1-mm translation or 1° rotation, and they were prohibited from this study. All participants mentioned previously fit the criteria without significant movements. After motion correction, data were normalized to the standard Montreal Neurological Institute (MNI) space and resampled to isotropic 3-mm voxels. Finally, the data were smoothed with a 6-mm full-width half-maximum Gaussian kernel to amplify the signal-to-noise ratio.

### Mean fractional ALFF map extraction

ALFF is critical physiological information in fMRI data and can be summarized as the magnitude of low-frequency fluctuations, which can represent cerebral activity in certain areas. The Resting-State Data Analysis Toolkit (version 1.8) was set to extract essential physiological information from preprocessed fMRI data [21]. ALFF was calculated with a band-pass filter of 0.01–0.12 Hz. The mean fractional ALFF (mfALFF) is a normalized mean ALFF, and therefore mfALFF can provide a more specific measure of low-frequency oscillatory phenomena than ALFF can.

Analysis of covariance (ANCOVA), multiple regression and post-hoc two-sample t tests were implemented to compare mfALFF among groups using SPM12. Participants’ age and education level were considered as covariates, and FDR-corrected p value of <0.05 was considered statistically significant.

### Graph theoretical analysis

Smoothed fMRI data were employed for graph theoretical analysis. The functional connectivity toolbox, CONN, simplified the connectivity matrix generation procedure [22]. CONN can calculate correlations voxel-to-voxel, but this can be time-consuming. To minimize the possible connections in the voxel space, we first segmented the whole brain of each subject into 90 regions based on the Automated Anatomical Labeling atlas [23], with each region considered a node [22, 24]. The connection between each node was viewed as an edge. The 90 × 90 connectivity matrix was then computed through Pearson correlations for each participant.

Graph Analysis Toolbox (GAT) accepted the output connectivity matrix of each group from CONN [25]. GAT was used to obtain the topological parameters, and the area under the curve (AUC) within chosen ranges for each topology index and comparing them between each group. Topological parameters were conducted in densities between 0.05 and 0.25, with an increment of 0.01. The density represents the ratio of existing connections to possible connections. A two-sample t test and nonparametric permutation test (×1000) was performed by GAT for statistical comparison.

Finally, the connectivity matrices generated from CONN were used for network-based statistical analysis (NBS). The NBS is based on the principles underpinning traditional cluster-based thresholding of statistical parametric maps [26]. The threshold was set for statistical tests in each edge, and the goal of using this method was to identify the alterations of cerebral subnetworks in group comparison.

## Results

### Demographic characteristics

Group comparisons were undertaken to investigate the differences between each group, and Table 1 summarizes the demographic characteristics of all participants. Age had statistical significance (p < 0.05) when comparing both the C+ and C- groups with the HC group. Mean education level also revealed an identical result as age. Furthermore, although the C- group scored highest in anxiety and depression, significance difference was noted only between the depression scores of the C- and HC groups.

**Table 1:** Demographic characteristics.

	C+ (N = 57)	C- (N = 45)	HC (N = 70)	p-value		
				A	B	C
Age (mean ± SD year)	50.1 ± 8.5	49.3 ± 9	43.3 ± 9.2	0.65	p<0.001	p<0.001
Range of age (year)	37–67	39–79	22–67			
Education year (mean ± SD year)	11.7 ± 3.7	12.3 ± 4.1	13.8 ± 3.1	0.4	p<0.001	0.03
Anxiety score (HADS)	3.9 ± 3.9	5.4 ± 4.5	4.1 ± 3.6	0.07	0.81	0.07
Depression score (PHQ-9)	3.9 ± 3.7	4.9 ± 4.2	3.1 ± 3.2	0.21	0.19	0.01

A: Comparison between C+ and C- group

B: Comparison between C+ and HC group

C: Comparison between C- and HC group

### mfALFF

**Fig 1:**
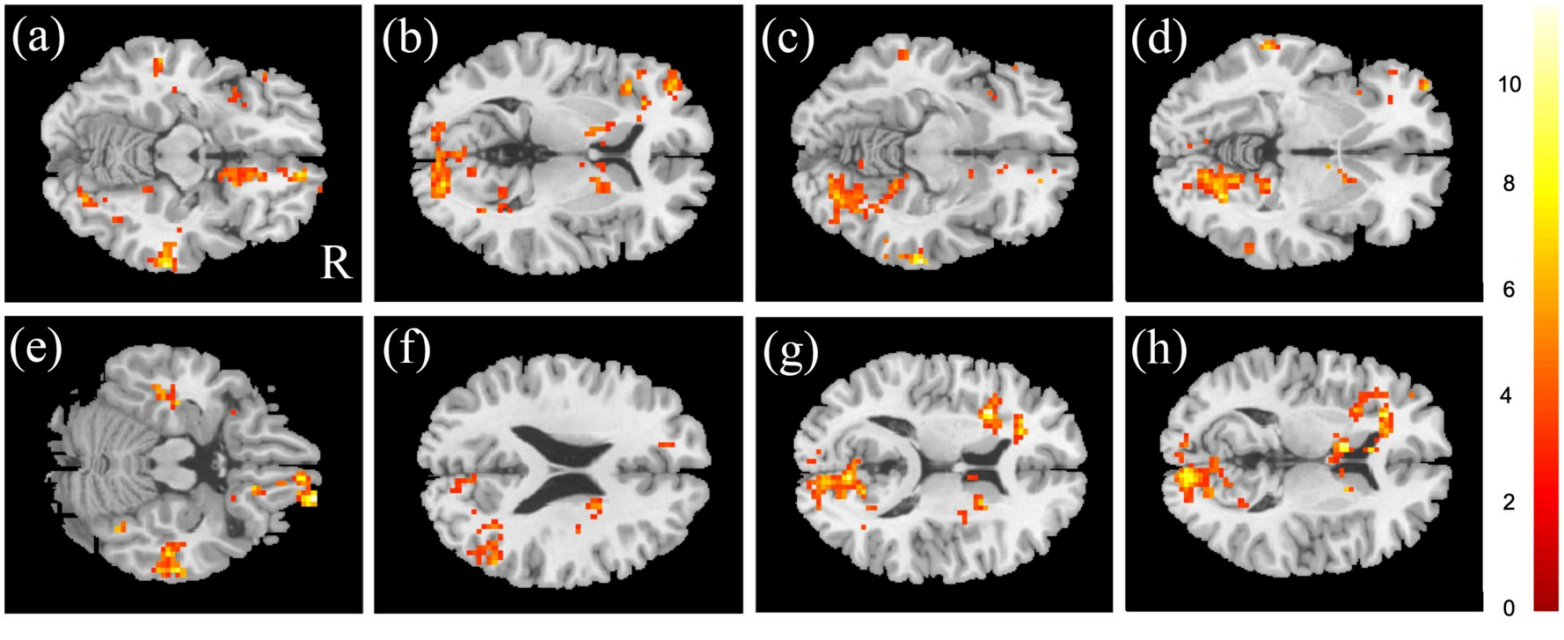
ANCOVA results of mfALFF in all three groups. Significant results were noted the (a) right superior frontal orbital gyrus, (b) left middle frontal gyrus, (c) right middle temporal gyrus, (d) left middle temporal gyrus, (e) right inferior temporal gyrus, (f) right angular gyrus, (g) left insula, and (h) left caudate (cluster size > 50, corrected p < 0.05, color bar, F scores).

**Fig 2:**
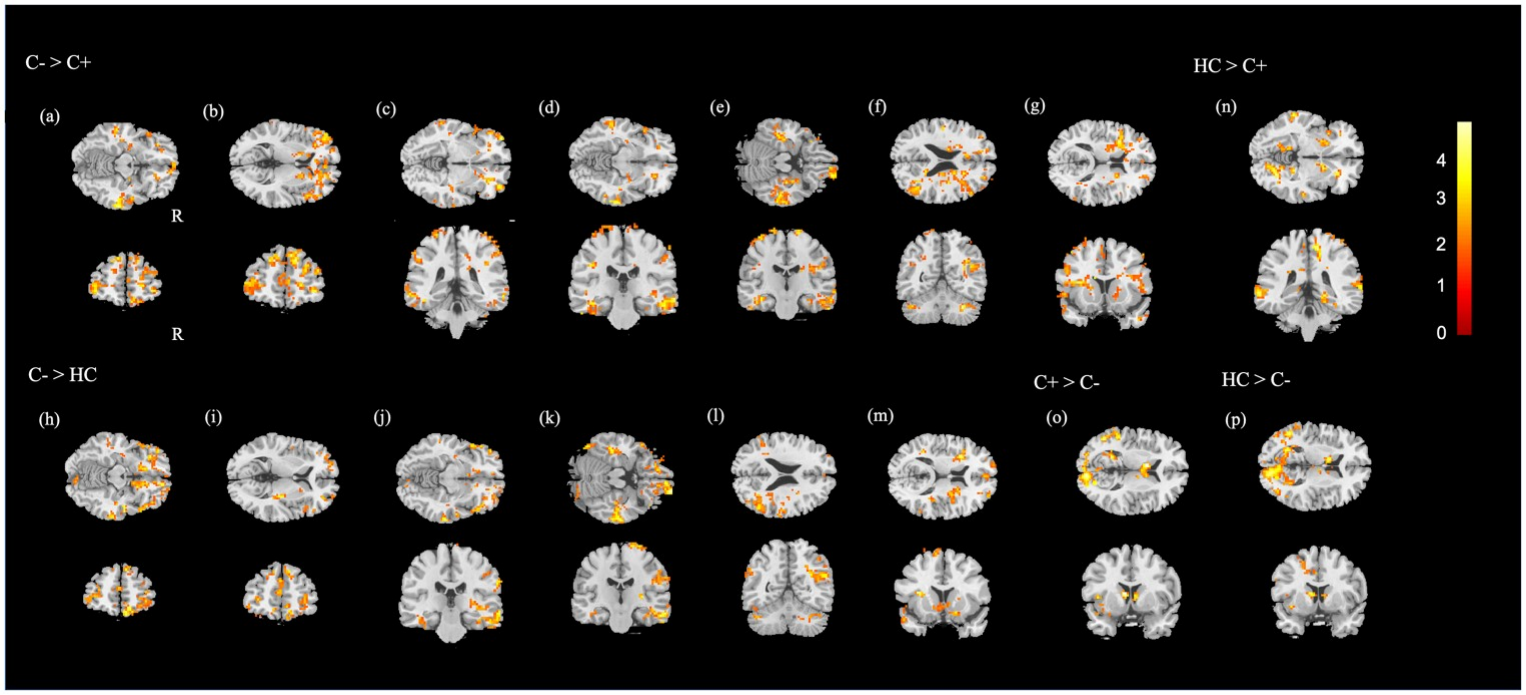
Post-hoc t test results. Compared with C+ group, hyperactivity on the (a, b) prefrontal cortex, (c, d) bilateral middle temporal gyrus, (e) right inferior temporal gyrus, (f) right angular gyrus and (g) left insula occurred in the C- group. Hypoactivity in HC group revealed in (h, i) prefrontal cortex, (j) right middle and (k) inferior temporal gyrus, (l) angular gyrus and (m) left insula in comparison between C- group as well. In all the post-hoc results (n) left middle temporal gyrus displayed hyperactivity in HC group than C+ group. Hypoactivity of the (o, p) left caudate in the C- group was revealed in comparison with the C+ and HC group (cluster size > 50, corrected p < 0.05, color bar, t scores).

ANCOVA performed to determine differences among the three groups revealed significant difference (p < 0.05) in the prefrontal cortex (PFC), bilateral middle, right inferior temporal gyrus, right angular gyrus, left insula, and left caudate (Fig 1). The post-hoc t test was performed for further evaluation to compare mfALFF in these areas. In the two-sample t test, mfALFF of the PFC, bilateral middle temporal gyrus, right inferior temporal gyrus, right angular gyrus and left insula were significantly higher in the C- group showed than C+ group (Fig 2a to 2g). Compared to C- group, the HC group demonstrated significantly lower activation in the PFC, right middle temporal gyrus, inferior temporal gyrus, angular gyrus and left insula (Fig 2h to 2m). For the left middle temporal gyrus, HC group revealed significantly greater than C+ group (Fig 2n). Furthermore, the left caudate, t tests indicated that the mfALFF of C- group was significantly lower than that of HC and C+ groups (Fig 2o and 2p).

**Fig 3:**
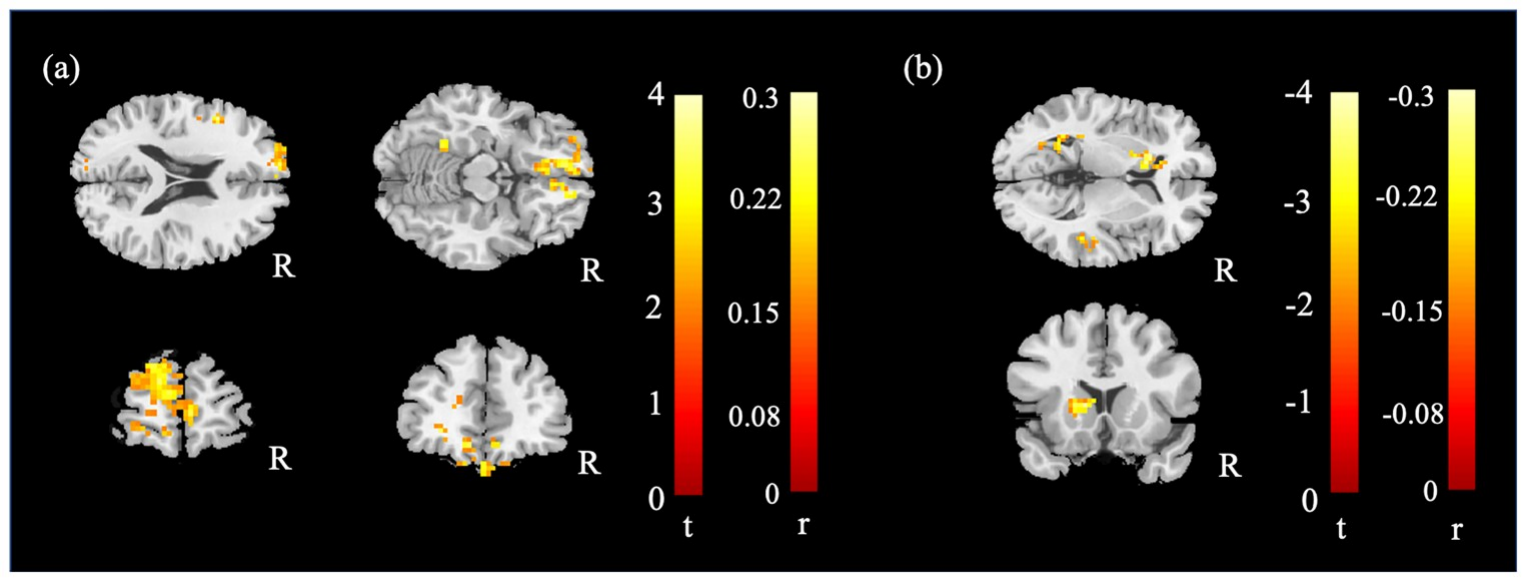
Multiple regression results between mfALFF and PHQ-9 score displayed that (a) positive correlation in PFC (b) negative correlation in left caudate (cluster size > 50, corrected p < 0.05, color bar, t scores, r scores).

Since PHQ-9 score was significantly higher in C- compared with HC group, multiple regression was applied to reach the correlation between mfALFF and PHQ-9 score. The given results showed positive correlation between prefrontal cortex and PHQ-9 score (Fig 3a) and negative correlation between left caudate and PHQ-9 (Fig 3b).

### Graph theoretical analysis

**Fig 4:**
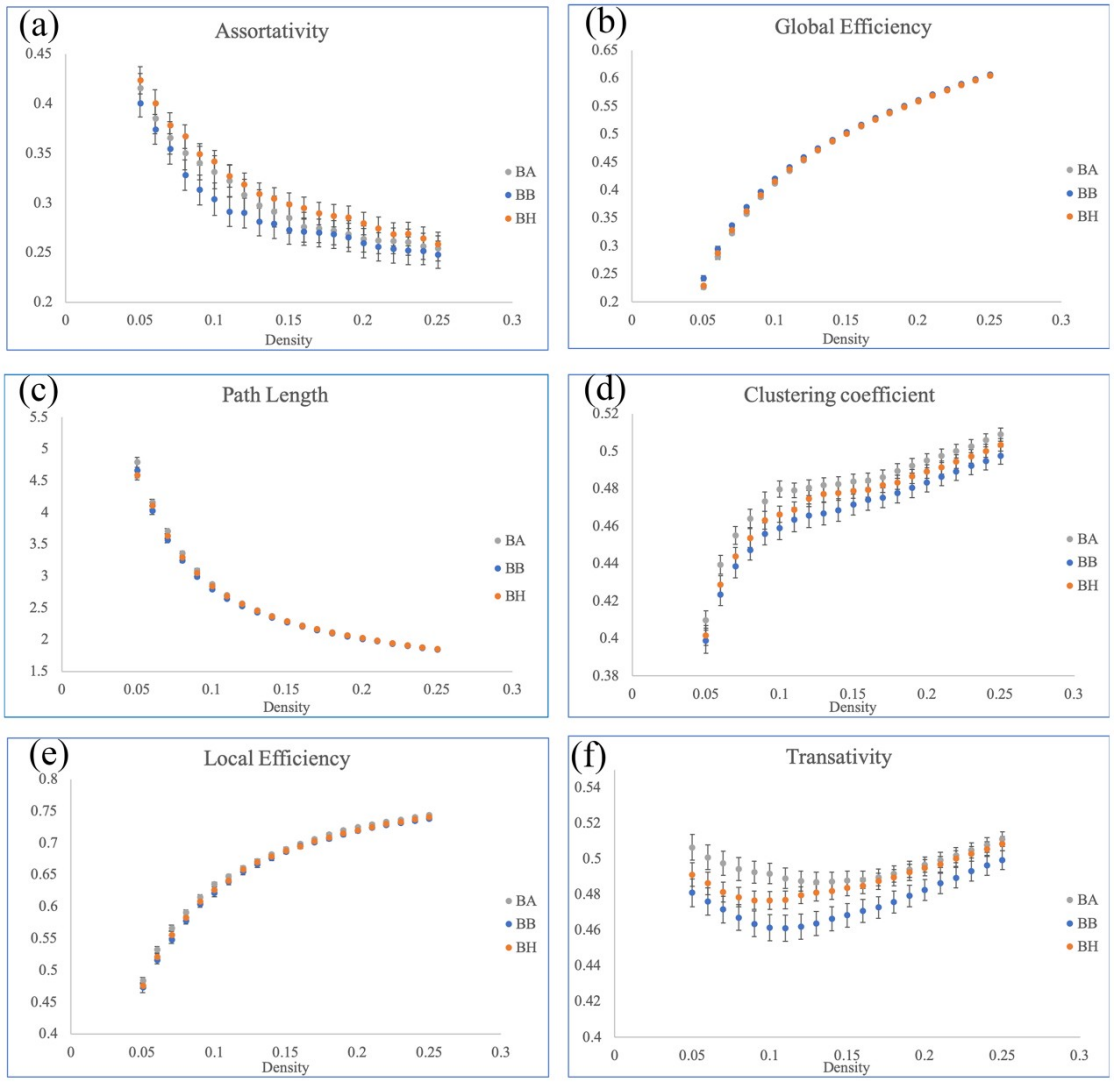
Topological parameters. (a) Both the cancer survivor groups demonstrated a decreasing trend in assortativity. Significant AUC was noted in the comparisons between the C+ and C- groups for (b) global efficiency, (c) characteristic path length, (d) clustering coefficient, (e) local efficiency, and (f) transativity. No significant differences was noted for AUC comparisons between both the cancer survivor groups and the control group.

**Fig 5:**
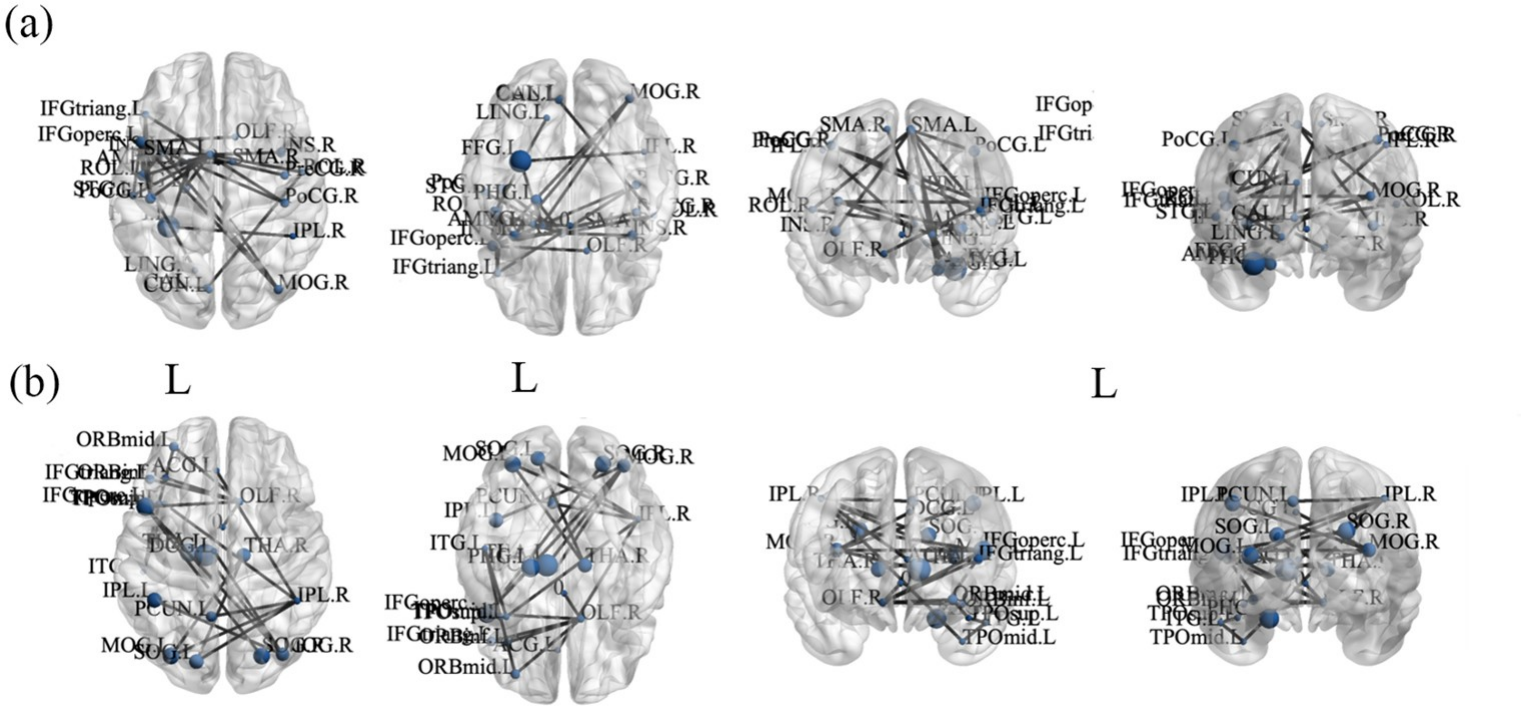
NBS results. (a) C+ group displayed significantly stronger subnetwork connections from the frontoparietal lobe to the subcortical nucleus and occipital lobe than did C- group (p = 0.05). (b) Although without statistical significance (p = 0.09), the HC group also showed stronger subnetwork connections on frontal, temporal, and occipital lobes than did the C- group.

For graph theoretical analysis, we used GAT for topological parameters extraction. Density was 0.05–0.25, and our results demonstrated subtle changes in the C+ and C- groups compared with the HC group. Both the C+ and C- groups demonstrated a decreasing trend in assortativity (Fig 4a) compared with HC group, but without significance based on AUC values. Compared with the HC group, the C+ and C- groups respectively demonstrated lower and higher global efficiency (Fig 4b) and longer and shorter characteristic path lengths (Fig 4c). The AUC comparison determined statistical significance when comparing C+ with C- group (p < 0.05). Furthermore, regarding the clustering coefficient, local efficiency, and transitivity (Fig 4d, 4e and 4f, respectively) demonstrated a decreasing and increasing trend in the C- and C+ group compared with the HC group, respectively. The AUCs for each index, except assortativity, demonstrated significant improvements in the C+ and C- groups (p < 0.05), but no significant difference was noted between the cancer survivor groups and the HC group. The overall trend indicated lower global integration abilities and greater local cliques for the C+ group. Greater global properties and declined local cliques in the C- group revealed that the C+ group inclined toward a regular network, whereas the C- group inclined toward a random network composition with a small-world index of >1. Furthermore, NBS results demonstrated that C+ group had significantly (p = 0.05) stronger connections from the frontoparietal lobe to the subcortical nucleus and occipital lobe than did C- group (Fig 5a). By contrast, although without statistical significance, the HC group demonstrated stronger subnetworks mainly from the frontal lobe to the temporal lobe than did the C- group (Fig 5b, p = 0.09).

## Discussion

This study explored functional and topological changes in individuals with breast cancer, with and without chemotherapeutical treatment. Previous studies have shown that breast cancer survivors often experienced posttreatment cognitive function impairment, such as affected memory, attention, processing speed, and word finding [3].

Cognitive impairment may also occur without receiving chemotherapy. The presence of cancer and stress, and cytokine levels are evaluated before administering chemotherapy. Cancer and surgery increase cytokine levels, which then impacts the brain [6, 27, 28]. In addition, the strong correlation between cognitive functions and stress level has long been studied. Increased stress levels disturb the hormone homeostasis of the body [27] and consequently affects the brain.

The C- group had hyperactivity in the PFC, bilateral middle temporal gyrus, right inferior temporal gyrus and right angular gyrus among the three groups. Only left caudates in C- group indicated hypoactivity than in other groups. Graph theoretical analysis demonstrated that the C+ and C- groups were inclined toward regular and random networks, respectively.

### Alteration in mfALFF

ALFF is defined as the magnitude of slow fluctuations that represent the activation of certain regions of the brain. The PFC is the part of the brain that controls cognitive skills, such as attention, execution, memory, and problem solving. Alteration in PFC has been proposed by previous studies [29, 30]. Our t test revealed that the C- group demonstrated greater activation of the PFC than did the C+ and HC groups. Although ANCOVA did not demonstrate significant results, several subregions in the frontal lobes in the C+ group also expressed higher mfALFF than did the HC group. Previous study revealed lower prefrontal activity in task based fMRI [31]. In our studies, we identified different pattern in rs-fMRI; statistical significance in depression scores was undiscovered between C+ and HC group, we suggest this phenomenon may reflect brain’s compensatory ability after chemotherapy. By contrast, the C- group demonstrated significantly higher depression scores than the other groups. The activity of the PFC is associated with depression, and both its subregions ventromedial PFC (vmPFC) and dorsolateral PFC (dlPFC) play significant roles in depression. Functional image studies have revealed that hyperactivity appears in the vmPFC and dlPFC during the progression of depression, and the contrary is true during the recovery phase. Changes in hemoglobin concentration may also correlate with depression [32].

Depression was also associated with the hypoactivity of the left caudate in the C- group. Our results indicated a significant decreasing mfALFF trend in the left caudate in the C- group. An event-based fMRI study revealed that both hippocampi and the anterior caudates contributed to learning ability [33]. Moreover, the anterior caudate is particularly associated with unstructured memory deficits in people with depression; this findings indicated that the caudate is critical in learning and forming memory [34]. Moreover, the bilateral caudate reduces glucose metabolism in prechemotherapy patients with depression, as noted under fluorodeoxyglucose positron emission tomography which is similar to our multiple regression result as well [35].

Here, the right angular gyrus, right middle temporal gyrus and right inferior temporal gyrus demonstrated significantly lower activity in the HC group than in the C- group. These regions are associated with declarative memory. Several studies have demonstrated that the middle temporal and inferior temporal gyri are involved in semantic memory, language, and sensory integration. Moreover, mental disorders might be associated with structural alterations in these gyri [36]. Structural alteration in the middle temporal gyrus is also associated with depression [37].

Each brain region has a specific function. However, these regions cannot completely account for all brain functions. Human cognition may be associated with multiple integrated regions [38, 39]. In human brain networks, certain regions have more connections than do others, and these connected regions are known as “hubs.” The angular gyrus connects hubs linked to various subsystems, and it may have several functions in the brain. A meta-analysis review of the angular gyrus summarized that the angular gyrus is considerably involved in attention mechanisms, particularly with shifting attention [40]. The angular gyrus is also associated with verbal working memory and episodic memory retrieval, with studies suggesting that the angular gyrus functions as an episodic memory buffer, carrying information until execution [41]. The angular gyrus is consistently recognized as crucial to default mode networks (DMNs), which link subsystems within the brain. People with depression also can display significantly greater ALFF than do those without depression, and thus, alteration on the right angular gyrus might be associated with the disturbance in the DMNs [42].

The insula, one of the least understood brain areas, serves as a network hub that integrates information across subregions. The insula is a popular subregion in cognitive neuroscience, and its role in decision-making, emotional processing, and attention has been studied [43]. Hyperactivity in the insula may be associated with fear and anxiety. Moroever, the insula is related to the central executive network (CEN), salience network, and DMN and is responsible for coordinating brain network dynamics and initiating the switch between DMNs and CENs [43].

### Changes of network measurement

Previous studies suggest that breast cancer and chemotherapy may altered brain network [44, 45]. In our study, the C+ and HC groups displayed identical topological parameter values. Other studies have examined cancer survivors after chemotherapy [46, 47] and observed symptomatic improvements 1 year after chemotherapy. These results have suggested that cognitive functions and symptoms self-recover with time. A possible reason for an insignificant C+ group result might have been our data collection bias: because patients who completed chemotherapy within 1 month were reluctant to receive fMRI scans, we recruited patients who had completed chemotherapy within 3–12 months. Thus, partial recovery may have already occurred in the C+ group when we recruited them into the study.

Changes in the local cliques can also be affected by mood. People diagnosed as having major depressive disorders demonstrated significant decreases in local connections [48]. The decreased local cliques could have been related to higher depression scores in the C- group than in the HC group. The alteration of network measures may be associated with working memory performance [49, 50]. Shifting network topology in the groups might have been connected to working memory performance. Global efficiency, local efficiency, and age may be associated with working memory performance [50]; however, this inference warrants further psychological evaluation.

The current results demonstrated that subregion connections, which indicate the ability of cerebral networks for parallel processes, were disrupted in both the cancer survivor groups. These groups demonstrated larger increases and decreases in the global integration than did the HC group. The elevated global integration and declined local clique in the C- group indicated that the functional connections were inclined toward random networks. The opposite trend in the C+ group suggested that patients who underwent chemotherapy were shifting toward regular networks, but small-world properties remained in both the cancer survivor groups (index values of both groups > 1).

Random networks are defined as those in which two given nodes have one edge; this network type displays high global integration. The hyperactivity of the functional hub presented in our results may have been associated with the high global integration of the C- group. However, the brain is an economic organ [7]; although two given nodes being connected appears to be efficient, random networks tend to cost much energy to maintain function. Regular networks are the opposite of random networks, in which nodes are connected to their nearest neighbors. Moreover, regular networks require less energy to maintain, but they are also less efficient. This topology has fewer direct connections and does not favor globally integrated information processing, and this may result in disrupted information when traveling across distant subregions. Moreover, reduced global efficiency contains a positive correlation to lower memory scores [51].

Overall, subtle changes appeared in both the cancer survivor groups than in the HC group, and the significant distinctions were evident in the comparison between the cancer survivor groups. Disturbances in the functional network organization might reflect reductions in the dynamic responses in both the cancer survivor groups. Our results suggested that alterations in functional networks occur with and without chemotherapy.

## Limitations

A potential limitation of our study is that we did not consider chemotherapeutic agent type [52] and dosage, cancer stage, and postmenopausal status. Future studies should overcome this limitation by including the aforementioned covariates.

## Conclusion

Here, functional connectome analysis was used to investigate functional changes among breast cancer survivors, and alterations were noted in various brain regions. Our results demonstrated subtle changes in both the cancer survivor groups and provided evidence that patients diagnosed as having breast cancer may have cerebral network alterations even before adjuvant chemotherapy initiation. Further longitudinal and experimental research should be conducted to confirm the mechanisms underlying these alterations.
